# Natural Bioactive Compounds Targeting NADPH Oxidase Pathway in Cardiovascular Diseases

**DOI:** 10.3390/molecules28031047

**Published:** 2023-01-20

**Authors:** Siti Sarah M. Sofiullah, Dharmani Devi Murugan, Suhaila Abd Muid, Wu Yuan Seng, Sharifah Zamiah Syed Abdul Kadir, Razif Abas, Nurul Raudzah Adib Ridzuan, Nor Hisam Zamakshshari, Choy Ker Woon

**Affiliations:** 1 Department of Anatomy, Faculty of Medicine, Universiti Teknologi MARA (UiTM), Sungai Buloh 47000, Selangor, Malaysia; 2 Department of Pharmacology, Faculty of Medicine, Universiti Malaya, Kuala Lumpur 50603, Malaysia; 3 Institute of Pathology Laboratory Medicine and Forensic Sciences (I-PPerForM), Universiti Teknologi MARA (UiTM), Sungai Buloh 47000, Selangor, Malaysia; 4 Department of Biochemistry and Molecular Medicine, Faculty of Medicine, Universiti Teknologi MARA (UiTM), Sungai Buloh 47000, Selangor, Malaysia; 5 Centre for Virus and Vaccine Research, School of Medical and Life Sciences, Sunway University, Subang Jaya 47500, Selangor, Malaysia; 6 Department of Biological Sciences, School of Medical and Life Sciences, Sunway University, Subang Jaya 47500, Selangor, Malaysia; 7 Department of Human Anatomy, Faculty of Medicine and Health Sciences, Universiti Putra Malaysia, Seri Kembangan 43400, Selangor, Malaysia; 8 Department of Anatomy and Embryology, Faculty of Medicine, Leiden University Medical Centre, 2333 ZC Leiden, The Netherlands; 9 Department of Chemistry, Faculty of Resources Science and Technology, University Malaysia Sarawak, Kota Samarahan 94300, Sarawak, Malaysia

**Keywords:** natural products, cardiovascular diseases, oxidative stress, NADPH signalling pathway

## Abstract

Cardiovascular disease (CVD) is the leading cause of death worldwide, in both developed and developing countries. According to the WHO report, the morbidity and mortality caused by CVD will continue to rise with the estimation of death going up to 22.2 million in 2030. NADPH oxidase (NOX)-derived reactive oxygen species (ROS) production induces endothelial nitric oxide synthase (eNOS) uncoupling and mitochondrial dysfunction, resulting in sustained oxidative stress and the development of cardiovascular diseases. Seven distinct members of the family have been identified of which four (namely, NOX1, 2, 4 and 5) may have cardiovascular functions. Currently, the treatment and management plan for patients with CVDs mainly depends on the drugs. However, prolonged use of prescribed drugs may cause adverse drug reactions. Therefore, it is crucial to find alternative treatment options with lesser adverse effects. Natural products have been gaining interest as complementary therapy for CVDs over the past decade due to their wide range of medicinal properties, including antioxidants. These might be due to their potent active ingredients, such as flavonoid and phenolic compounds. Numerous natural compounds have been demonstrated to have advantageous effects on cardiovascular disease via NADPH cascade. This review highlights the potential of natural products targeting NOX-derived ROS generation in treating CVDs. Emphasis is put on the activation of the oxidases, including upstream or downstream signalling events.

## 1. Introduction

Cardiovascular disease (CVD) remains the major cause of mortality and premature death worldwide [1]. In 2019, according to the World Health Organization, 17.9 million deaths were reported from cardiovascular disease, accounting for 32% of all global fatalities, and 85% of these deaths are the result of a heart attack or a stroke [2]. It has been reported that 58% of the 17.9 million CVD fatalities that occurred globally were in Asia [3]. Risk factors for cardiovascular disease include sedentary lifestyle, unhealthy diet, smoking, diabetes, age, and genetics [4]. These risk factors unite behind a convergence of mechanisms involving reactive oxygen species (ROS), as reported in vitro, in vivo, and clinical studies [4]. Oxidative stress is a result of a change in redox equilibrium that leads to an imbalance between ROS formation and endogenous antioxidant systems [5]. Oxidative stress elicits deleterious effects by inducing damage to macromolecules, such as deoxyribonucleic acid (DNA), ribonucleic acid (RNA), protein, and lipids. The imbalance in ROS metabolism has been reported in the pathogenesis of several CVDs, including heart failure, stroke, hypertension, atherosclerosis, etc. [6]. In fact, oxidative stress is no longer focused on the imbalance between ROS production and scavenging, but on the relevant enzyme dysfunctions [7]. In biological systems, several major factors that contribute to the production of ROS are an increase in mechanoreceptor activation [8], mitochondrial electron transport chain, lipoxygenases, cytochrome P450 oxidases, nitric oxide (NO) synthase, xanthine oxidase (XO), and nicotinamide adenine dinucleotide phosphate (NADPH) oxidase [6]. Among these, NADPH oxidase (NOX) is the primary source of ROS in the cardiovascular system, which affects a variety of signalling pathways [9,10,11] and certain transcription factors [12] to modify cardiovascular cell function. 

Statins and metformin are examples of prescribed drugs that function as antioxidants, scavenging free radicals and ROS superoxide (O_2_^−^) in CVD [13]. In order to overcome undesirable side effects or any harmful effects of prescribed cardiovascular drugs with prolonged usage [14], research is shifting gears towards natural products due to their greater safety and potent various medicinal properties, including antioxidant [15]. Natural products, such as herbs and spices, are the major source of development of active drugs and are used in traditional medicine [15]. Furthermore, the preparation of these natural products is easier and low cost [16]. This is due to the availability of these natural products, which can be sourced locally and are easily available throughout the year [16]. In addition, due to the low toxicity beneficial properties of natural products, they have long been used as therapeutic agents to combat cardiovascular disease [17]. Thus, this review is aimed at highlighting natural products which are being used against cardiovascular disease by inhibiting NADPH signalling pathways. We hypothesise that natural products are effective in inhibiting specific subunits of NADPH in cardiovascular conditions, such as cardiac damage, arteriosclerosis, hypertension, aortic valve disease, myocardial ischemic-reperfusion injury, hypercholesterolemia, and cardiac hypertrophy.

## 2. Target Pathway: NADPH Oxidases

### 2.1. The NOX Family of NADPH Oxidases

NADPH oxidases (NOXs) are a family of multi subunit enzymes that produce superoxide anion radical (O_2_^−^) by oxygen reduction using NADPH or nicotinamide adenine dinucleotide (NADH). They are found in phagocytic cells (neutrophils, monocytes, and macrophages) and non-phagocytic cells, such as vascular smooth muscle cells (VSMC) and endothelial cells [18]. NOXs are also implicated in redox-sensitive signalling pathways that are crucial for physiological cellular functions, including differentiation, growth, and proliferation [19,20]. NOXs are acknowledged as the primary generator of ROS in cells, along with a respiratory chain’s mitochondrial enzymes. Other than function of cell signalling and innate immune response, numerous diseases, including cancer, neurodegeneration, and muscular dystrophy, are exacerbated by these enzymes [21]. NOX has the qualities of a second messenger other than its responsibilities in host defence; the production of ROS by NOXs is highly regulated, and while NOXs are not ubiquitous, they are specified in certain membrane tissue and compartments. Superoxide is produced when NOXs transfer electrons from NADPH to heme groups, flavin adenine dinucleotide (FAD), and eventually to molecular oxygen. Genetic instability is associated with high amounts of ROS produced by exudative NOX activity, followed by proliferative senescence, DNA damage response activation, hyperproliferation, and occasionally, apoptosis [22]. NOXs are important in the pathophysiology of many CVDs. They have been recognised for their complexity, regulation, and specific function in the underlying molecular mechanisms of the pathogenic processes in CVDs [6].

Seven components have been identified; based on the isoform, they are produced in various cardiovascular signalling pathways and cell compartments and control a variety of processes, including inflammatory responses, migration, proliferation, differentiating, senescence and apoptosis. Each isoform contains up to seven regulatory subunits in addition to a catalytic subunit, known as NOX (NOX-1–5) or dual oxidase (DUOX); (DUOX–1-2, also known as NOX–6-7) [23]. NOX1, NOX2, NOX4, and NOX5 have been shown to have a considerable expression in the cardiovascular system, which will be the focus of this review (Table 1).

#### 2.1.1. NOX1

NOX1 was discovered to be the first homolog of NOX2 and shared 60% amino acid similarity [23,24,25]. Despite the lack of information on NOX1 subcellular location, it has been hypothesised that this protein is membrane-localised and may be found in caveolar rafts [26]. NOX1 is broadly expressed in various cell types, with a particularly high level of expression in the colon epithelium [27,28]. The identification of NOX organiser 1 (NOXO1), a homolog of cytosolic protein p47phox, and NOX activator 1 (NOXA1), a homolog of cytosolic protein p67phox, provided evidence that NOX1 action is dependent on cytosolic subunits [29,30]. NOX1 requires the p22phox (membrane subunit), cytosolic subunits, and the Rac guanosine triphosphatase (GTPase) to be activated [31,32]. In short, the combination of several subunits is necessary for NOX1 activation [33] where it required p47phox, which then is transported to the membrane in which it binds to p22phox [33].

#### 2.1.2. NOX2

NOX2 was the first enzyme from the NOX family to be identified [33]. NOX2, also known as gp91phox, has six transmembrane domains, with the C- and N-termini facing the cytoplasm. Furthermore, NOX2 is stabilised by constitutive interaction with p22phox [34,35]. NOX2 activation needs translocation and interaction with phosphorylated p47phox in order for other cytosolic components, such as p67phox and p40phox, to attach to the NOX2/p22phox complex [36,37,38]. Upon complex formation, the Rac GTPase first connects with NOX2 and then with p67phox, resulting in an active complex for superoxide generation via electron transfer from cytosolic NADPH to oxygen on the luminal or extracellular area [39,40].

#### 2.1.3. NOX4

Geiszt et al. discovered that NOX4 has 39% sequence homology with NOX2 [41,42]. NOX4 activity is substantially influenced by p22phox [43], but not by cytosolic subunits [44]. Furthermore, the role of Rac in NOX4 activation remains debatable [45]. Polymerase delta-interacting protein 2 (Poldip2), a polymerase (DNA-directed) delta-interacting protein, acts as a positive regulator in vascular smooth muscle cells by interacting with NOX4/p22phox [46]. The connection of Poldip2 with p22phox was demonstrated by using glutathione S-transferase (GST) pull-down assays [46]. The use of Western blotting, real-time quantitative reverse transcription PCR (qRT-PCR), and immunohistochemistry on NOX4-rich tissues (e.g., aorta, lung, and kidney) revealed enhanced Poldip2 expression [46,47]. The role of Poldip2 as a positive regulator of NOX4 in conjunction with p22phox has been established in vascular smooth muscle cells utilising short interfering RNA (siRNA) against Poldip2 [46].

#### 2.1.4. NOX5

NOX5, a protein with five isoforms, was found by two separate research groups. Banfi et al. [48] found isoforms a, b, c, and d, while Cheng et al. [49] discovered a fifth isoform, NOX5e or NOX5-S. NOX5 isoforms a-d have a lengthy, intracellular N-terminal domain with a Ca21-binding EF-hand region [49,50], but the fifth isoform lacks the EF-hand region and is structurally identical to NOX1-4 [49]. NOX5 isoforms a-d are activated by cytosolic calcium rather than p22phox or cytosolic subunits, indicating the presence of a Ca21-binding domain [51]. In contrast, the NOX5e isoform, which lacks a Ca21-binding domain, relies on the cAMP response element binding protein to function [52]. The N-terminal calmodulin-like domain with four Ca^2+^ binding sites makes NOX5 distinct from other NOX isoforms. NOX5 stimulation is Ca^2+^-dependent and independent of engagement with designated subunits. The fundamental structure of DUOX1 and DUOX2 is similar to NOX5; however, they are linked to an extracellular N-terminus and transmembrane domain [33].

**Table 1:** NOX family and its regulatory subunit and expression in various tissues or cells.

NOX Family	Regulatory Subunit	Expression Observed in			
		Tissue	Reference	Cells	Reference
1	NOXO1, NOXA1, p22phox, Rac	Smooth muscle Colon epithelial Uterus and placenta	[53] [54] [55]	Endothelial cells	[56]
				Cardiomyocytes	[57]
				Vascular smooth muscle cells	[58]
				Human aortic smooth muscle cells	[59]
				Smooth muscle cells	[60]
2	p22phox, p47phox, p40phox, and p67phox, Rac	Heart tissue	[61]	Endothelial cells, adventitial cells	[62]
				Cardiomyocytes, smooth muscle cells of the arteries	[63]
				Coronary microvascular endothelial cells	[64]
				Human umbilical vein endothelial cells	[65]
4	p22phox, Poldip2	Lung tissues Ovary and eye Fetal tissues Kidney	[66] [67] [49] [68]	Endothelial cells	[69]
				Cardiomyocytes, adventitial cells	[70]
				Vascular smooth muscle cells	[71]
				Human aortic smooth muscle cells	[72]
				Mesangial cells	[73]
				Hepatocytes	[74]
5	calmodulin-like domain with four Ca^2+^	Fetal, spleen and uterus tissues Lung, heart, thymus, liver, kidney, skeletal muscle	[49] [75]	Vascular smooth muscle cells Cardiomyocyte	[76] [70]

### 2.2. Expression of NOX Isoforms-Derived ROS in CVD

Numerous pathways related to CVD are associated with NADPH oxidase-derived ROS produced by vascular and phagocytic cells. The vasculature expresses certain NADPH oxidases in a constitutive manner [77]. In CVD, imbalanced ROS production causes vascular injury by stimulating several processes, such as vascular cell proliferation, migration, the deposition of extracellular matrix (ECM) proteins, or inflammation, sequentially enhancing vascular remodelling. The NADPH oxidase family regulates vasculature remodelling and is a significant generator of ROS in the arterial wall during CVD.

In various CVDs, dysfunctional mitochondria are believed to be the intracellular producer of ROS. It has been demonstrated that NOX-derived ROS can reach the mitochondria and induce electron leakage and the formation of mitochondrial ROS, indicating that defective mitochondria are connected downstream of NOXs [33]. On the other hand, eNOS uncoupling activity is also modulated by mitochondrial ROS production. Peroxynitrite, a harmful radical formed when the O_2_^−^ interacts with NO, can damage mitochondria by oxidising membrane lipids and electron transport chain complexes, resulting in defective cardiac contractile activity and impaired cardiac mitochondrial function [33]. Additionally, research has shown xanthine oxidase (XO) as a contributor of ROS in developing a range of CVDs. The enzyme xanthine oxidoreductase is first produced in the dehydrogenase form (xanthine dehydrogenase; XDH), which is then quickly turned by oxidation into the oxidase form (XO) [78]. The ROS, O_2_^−^, and hydrogen peroxide (H_2_O_2_) are produced via XO by directly delivering electrons to molecular oxygen (O_2_) through one-electron and two-electron reductions, respectively [79].

In general, different NOX subunits are related to various pathophysiology of CVD. NOX1 and NOX4 play a role in the remodelling of the vascular system in various clinical situations, including atherosclerosis, pulmonary hypertension, restenosis aortic stenosis, and hypertension [58]. NOX1, NOX2, and NOX4 are expressed in endothelial cells, vascular smooth muscle cells exhibit NOX1, NOX4, and NOX5, cardiomyocytes express NOX1, NOX2, NOX4, and NOX5, whereas NOX2 and NOX4 are mainly expressed by adventitial cells [33]. NOX1-derived ROS promote vascular remodelling upon triggering vascular smooth muscle cell dedifferentiation, as well as promoting its migration and proliferation with other activities, such as the deposition and remodelling of ECM proteins. Although NOX4 is shown to play a part in migration and proliferation of VSMC, it has also been demonstrated to maintain the smooth muscle phenotype. The role of NOX4 is debatable, but NOX1 plays a harmful part in vascular remodelling in several diseases. Therefore, NOX4 may have advantageous or detrimental effects based on the disease model or the specific cell site.

Studies using cell-specific NOX deletion will clarify the precise function of each NOX isoform, particularly in vascular remodelling models [77]. NOX1 and NOX2 overexpression has been shown in studies to promote inflammation and oxidative stress, which can lead to cardiologic conditions [80,81]. NOX4 has an impact on a variety of cellular processes related to vascular remodelling, including cell proliferation, apoptosis, senescence, cell differentiation, cell migration, and cell cycle regulation [46,82,83]. NOX4 expression has been found to increase in the heart in response to pressure overload induced by transverse aortic constriction (TAC) over 2–4 weeks, as well as after phenylephrine or angiotensin II infusion [84,85,86,87]. NOX5 is involved in platelet-derived growth factor (PDGF) and capillary-like structure formation. NOX5 downregulation has been shown to reduce the production of thrombin-stimulated growth factor and tube formation. In contrast, NOX5 upregulation increased the endothelial nitric oxide synthase (eNOS) activity, but decreased NO bioavailability [88]. Given these, NOX5 overexpression disrupts the acetylcholine-induced relaxation and phenylephrine-induced contractile effects [81,88,89,90].

There is evidence that angiotensin II (Ang II) is crucial in the development of cardiovascular disorders linked to hypertension. In cultured human aorta smooth muscle cells, angiotensin II increased the expression of the NOX subunit’s mRNA for NOX1, NOX4, p67phox, p47phox, and p22phox [59]. This finding raised the possibility that NOX1 and NOX4 are involved in the oxidative stress that angiotensin II causes in human vascular smooth muscle cells. Moreover, angiotensin II enhanced the protein expression of NOX subunits (NOX2, p22phox, p47phox, p40phox, and p67phox) in smooth muscle cells of the arteries obtained from gluteal biopsies of healthy individuals. 

**Figure 1:**
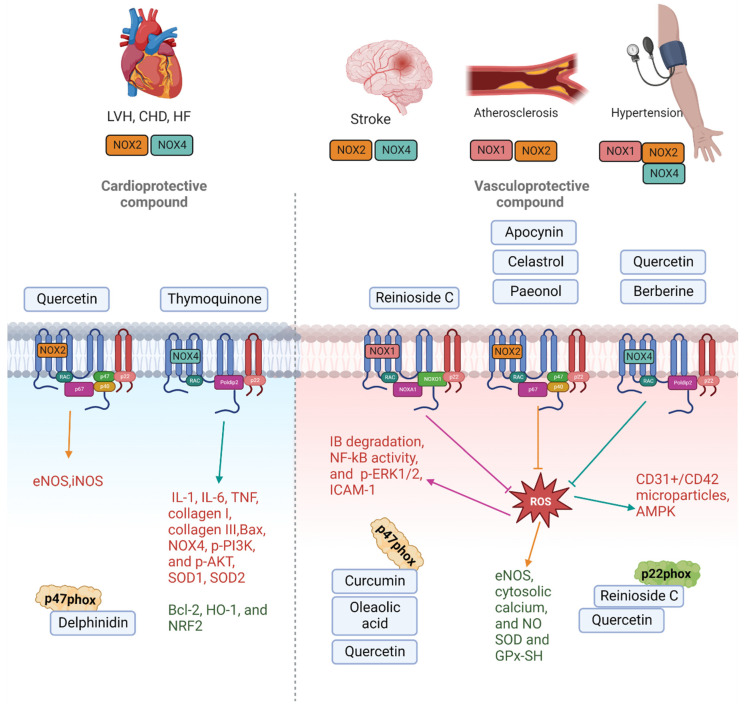
Schematic diagram of the NOX isoform-derived ROS production in cardiovascular diseases and natural products targeting those NOX isoforms leading to changes in intracellular targets. Natural products are divided into cardioprotective compounds targeting NOX2, NOX4, and p47phox, and vasculorprotective compound targeting NOX1, NOX2, NOX4, p47phox, and p22phox. The reduction in intercellular protein expression is noted in red font, while the upregulation of proteins is noted in green font in the diagram.

In atherosclerotic lesions of apolipoprotein E-deficient (*ApoE*/) mice and humans, the literature findings showed that expression of the NOX1 and/or NOXA1 subunits increased, suggesting that the enzyme plays a role in this disease state [91] (Figure 1). There is evidence that Nox1/*ApoE*/double knockout mice have a higher reduction in the formation of atherosclerotic lesions in the aortic arch than *ApoE*/single knockout animals [92]. NOX2 appears to be involved in the redox signalling involved in the start and progression of atherosclerosis. Overexpression of NOX2 in *ApoE*/mice boosted macrophage recruitment, but did not affect atherosclerosis progression [93].

Furthermore, a previous study showed that global NOX2 deletion mice increased activity of NADPH oxidase isoforms contributes to worsening outcomes following stroke, at least in males [94], and administration of apocynin before cerebral ischaemia improves outcome in wild-type, but not NOX2-deficient mice, suggesting apocynin’s protective effects occur via NOX2 inhibition (Figure 1) [94]. Neuronal apoptosis and blood–brain barrier leakage, which are pathophysiological characteristics of ischemic stroke, are caused by NOX4-mediated oxidative stress and leads to neuronal damage [95]. Neither sex of NOX4-deficient mice was largely protected against oxidative stress, blood–brain barrier leakage, and neuronal death after temporary and permanent cerebral ischemia. However, NOX1 and NOX2-deficient mice did not show this protection.

In heart failure, ROS regulate fibroblast proliferation, collagen synthesis, and matrix metalloproteinase activation, resulting in cardiac hypertrophy, fibrosis, and necrosis, which can lead to endothelial and myocardial dysfunction [96]. When pressure overload was induced by constriction of the ascending aorta in NOX2-deficient mice, a large increase in messenger (m)RNA expression of p22phox and a slight increase in mRNA expression of p47phox was reported, whereas, in wild-type mice, there was a moderate increase in p22phox and no increase in p47phox. This was reflected by the ROS levels in the myocardium, which was twice as high compared to wild-type animals. Similarly, NOX4 expression was raised in cardiomyocytes in response to pressure overload and after myocardial infarction in NOX4−/− mice and a cardiomyocyte-targeted NOX4 transgenic model. NOX4−/− mice, on the other hand, exhibited much greater cardiac dilatation and contractile degradation than wild-type mice, but NOX4 transgenic mice had less hypertrophy and fibrosis than wild-type mice. These suggest that the NOX2 and NOX4 isoform and the subunits of the enzyme complex associated with it were identified as a critical source of ROS in pressure-induced left ventricular hypertrophy, and they contributed to pathophysiologic alterations such as redox-sensitive kinase activation and heart failure development (Figure 1).

Using NOX isoform-specific knockout (KO) mice, researchers studied the activities of endogenous NOX1, NOX2, and NOX4 in myocardial ischemic-reperfusion (I/R) damage. Systemic NOX1, NOX2, and NOX1/NOX2 double KO mice showed a significant reduction in myocardial infarct size following I/R, while systemic NOX4 KO mice did not [97]. Furthermore, in response to I/R, cardiac-specific NOX4 transgenic mice showed increases in ROS generation and infarct size [39]. These data imply that NOX2 and NOX4 play a role in myocardial I/R damage (Figure 1).

## 3. Natural Products Targeting NADPH Oxidase Pathway in Cardiovascular Disease

The use of medicinal herbs as an alternate treatment option has gained an incredible interest in modern medical systems, especially in CVDs. This review provides a comprehensive summary of the published scientific data on the regulation of NADPH oxidase pathways and its related molecular mechanisms of natural bioactive compounds in its therapeutic approach for the prevention and treatment of various CVDs experimental models (Table 2).

### 3.1. Berberine

Berberine or 5,6-dihydro-9,10-dimethoxy-benzo[g]-1,3-benzodioxolo [5,6-a]quinolizinium is found as an active component in the roots, stem bark, and rhizomes of *Hydrastis canadensis* (goldenseal) plants [98]. Berberine has been used as an Ayurvedic and Chinese medicine for over 3000 years due to its potent antimicrobial, antiprotozoal, and antidiarrheal properties [99]. Berberine is recognised as one of the most promising natural compounds for treating metabolic illnesses, such as hyperlipidemia [100], obesity [101], gout [102], non-alcoholic fatty liver disease (NAFLD) [103], and type 2 diabetes [104].

Cheng et al. demonstrated berberine improves endothelial function in human study and in vitro human umbilical vein endothelial cells (HUVECs) by reducing endothelial microparticle (EMP)-mediated oxidative stress [105]. In this study, healthy middle-aged people who received berberine therapy for one month demonstrated increased endothelium-dependent vasodilation, but not endothelium-independent vasodilation measured by flow-mediated vasodilation (FMD) and sublingual nitroglyceride-mediated vasodilation. Additionally, after berberine treatment, serum MDA and circulating CD31+/CD42 microparticles were considerably reduced. The same research group also discovered that pre-treatment with the antioxidants berberine and apocynin decreased NOX4 protein expression in HUVECs, while endothelial microparticles enhanced it. These findings suggest that NOX4 may play a significant role in enhancing EMP-mediated ROS generation, resulting in decreased NO bioavailability and endothelial cell damage, which can be reversed by berberine treatment.

In another study, Zhang et al. demonstrated that by increasing eNOS expression and decreasing NOX4 expression, berberine reduces palmitate-induced endothelial dysfunction, and this regulatory impact of berberine may be connected to the activation of AMP-activated protein kinase (AMPK) [106]. In cardiovascular cells, AMPK plays a significant role as an NADPH oxidase inhibitor. When activated, AMPK lowers ROS production by reducing NADPH oxidase activity, which, in turn, prevents the endothelial cell death caused by palmitic acid in cultured HUVECs. Berberine treatment activated eNOS and inhibited NOX4-derived ROS accumulation in endothelial cells, which will help reduce oxidative stress through activation of AMPK, which may help explain why berberine has protective effects on endothelial function. Berberine significantly increased AMPK and p-AMPK protein expression levels in palmitate-induced endothelial dysfunction in HUVECs; however, Akt and p-Akt protein expression was unchanged. The study suggests that berberine may be a novel therapeutic medication for the prevention and treatment of CVD, warranting further clinical research.

### 3.2. Paeonol

*Paeonia suffruticosa* has a variety of medicinal properties and has been used for thousands of years in traditional oriental medicine [107]. The main component isolated from the root bark of *Paeonia suffruticosa* is paeonol [107]. Paeonol, also known as 1-(2-hydroxy-4-methoxyphenyl) ethenone, is insoluble in water [108]. Paeonol has been shown in numerous in vitro and in vivo studies to be an antiinflammatory compound [109,110,111]. Paeonol has been linked to neuroprotection in diabetic encephalopathy [112], Parkinson’s disease [113], and Alzheimer’s disease [114].

In mice induced by tunicamycin, chronic treatment with paeonol protected endothelial function and stabilised blood pressure by inhibiting endoplasmic reticulum (ER) stress-associated reactive oxygen species (ROS) [115]. Tunicamycin (1 mg/kg/week) was injected intraperitoneally into male C57BL/6J mice for two weeks to induce ER stress. Their study demonstrated that tunicamycin-treated mice had higher blood pressure, loss of weight, and had impaired endothelium-dependent relaxations of the aorta, all of which were improved by co-treatment with paeonol, TUDCA (ER stress inhibitor), or tempol (antioxidant). Co-treatment with paeonol or tempol reduced tunicamycin-stimulated up-regulation of NADPH subunits, NOX2, and nitrotyrosine (marker for peroxynitrate, an indication of enhanced oxidative stress) in mice, compared to the control group as demonstrated via Western blot. An increased ROS formation in *en face* endothelium and O_2_^−^ level was also observed in mice treated for 2 weeks with tunicamycin compared to the control group, as reflected by the intensity of DHE fluorescence staining and lucigenin-enhanced chemiluminescence (LEC). Additionally, chronic paeonol, TUDCA, and tempol treatment increased the phosphorylation of eNOS at Ser1176 in the aortas, whereas tunicamycin treatment decreased it. Thus, paeonol enhanced nitric oxide bioavailability in mouse aorta via inhibition of ROS, specifically on NOX2 [115].

Methotrexate (MTX) is known to increase reactive oxygen species (ROS) by increasing homocysteine, which is rapidly autoxidized and activates NOX, which generates ROS. In another separate study, paeonol reduced methotrexate (MTX)-induced cardiac damage in rats by decreasing NOX2 levels, increasing glutathione (GSH) levels, and elevating superoxide dismutase (SOD) activity [116]. Finally, paeonol protects against MTX-induced cardiac damage by decreasing oxidative and nitrosative stress, which, subsequently, suppressed the toll-like receptor 4 (TLR4) inflammatory pathway and also restored the histological structure.

These findings add to the growing evidence that paeonol could be used as a novel therapeutic drug or health supplement for patients with ER-stress-related cardiovascular disorders and methotrexate (MTX)-induced cardiac damage.

### 3.3. Thymoquinone

Thymoquinone (TQ) is a monoterpene compound identified chemically as 2-methyl-5-isopropyl-1, 4-benzoquinone and is the most prominent constituent of Nigella sativa seeds essential oil [117,118]. TQ has been studied for a wide range of pharmacological activities, which include antioxidant [119] anti-inflammatory [120], immunomodulatory [121], antihistamine [122], antimicrobial [123], and antitumour properties [124].

Zhang et al. reported thymoquinone protects apolipoprotein E-deficient (*ApoE*−/−) mice against angiotensin II (Ang II)-induced heart injury [125]. The *ApoE*−/− mice treated for 4 weeks with Ang II and TQ demonstrated a decreased area of cardiac fibrosis from Masson trichrome-stained sections compared to those treated with Ang II alone. This was further supported by reduced collagen I and III expression in the heart tissue Ang II-treated mice given TQ. Gene and protein expression studies showed elevated pro-inflammatory cytokines, tumour necrosis factor (TNF)- α, interleukin (IL)-1β and IL-6 in Ang II group, while TQ treatment suppressed this increase. Additionally, TQ decreased the expression of NOX4 and p53 in the cardiac tissue compared to those treated with Ang II alone. This group also showed NOX4, p53, collagen I and III, IL-1, IL-6 and TNF- α, protein expression levels were increased when in rat cardiac H9c2 cells treated with Ang II, which were reversed by TQ. Therefore, these results suggested that TQ could protect the heart from Ang II-induced injury suppressing oxidative stress, particularly NOX4.

Moreover, Guo et al. demonstrated that TQ inhibited inflammatory cell infiltration, the production of pro-inflammatory cytokines, apoptosis, oxidative stress, and PI3K/AKT pathway activation, all of which are indicators of sepsis-induced cardiac damage [126]. Four groups of male BALB/c mice were created randomly: the control, TQ, cecal ligation and puncture (CLP), and CLP + TQ groups. Following two weeks of TQ gavage on the mice, CLP was carried out. Histological alterations in the cardiac tissue after 48 h were evaluated. Markers linked to apoptosis, oxidative stress, inflammation, and the PI3K/AKT pathway were quantified. Compared to the control group, there were increases in NOX4 expression and decreases in Heme Oxygenase-1 (HO-1) and nuclear factor erythroid 2–related factor 2 (Nrf2) expression in the CLP group. However, NOX4 overexpression was normalised by TQ administration, followed by HO-1 and Nrf2 downregulation. In addition, TQ reversed the increase of IL-6, TNF- α, Bax, p-PI3K, Bcl-2, and p-AKT as well as decreased intestine histological changes. According to these findings, TQ is crucial in the treatment of sepsis-induced cardiac damage because it efficiently inhibited NOX4 mainly via PI3K/AKT pathways.

Furthermore, Chen et al. reported that through the activation of AMPK and suppression of MAPK signalling in vivo and in vitro, TQ has a protective impact on pathological heart hypertrophy [127]. Transverse aortic constriction (TAC) or a sham procedure was performed on male C57BL/6J mice, and both procedures were followed by six weeks of TQ therapy. Neonatal rat cardiomyocytes (NRCMs), used in in vitro investigations to stimulate cardiomyocyte hypertrophy, were treated to phenylephrine (PE) stimulation. The result also showed that ventricular tissues with 6 weeks of pressure overload had much higher ROS levels, while tissues treated with TQ had levels that were nearly identical to those of the group that underwent sham surgery. TQ improved the mRNA expression of genes associated to oxidative stress (NOX4, SOD1, and SOD2) in mice from the TAC group. Thus, TQ was shown to be a potential drug for the treatment of cardiac hypertrophy, since it protects against the condition by reducing NOX4 in an AMPK-dependent way.

### 3.4. Reinioside C

Reinioside C is the primary component extracted from *Polygala fallax Hemsl.* [128]. *Polygala fallax Hemsl*. is a Polygalaceae genus shrub or small tree that grows in the shade and humid environment of the valley forest and is primarily distributed in Jiangxi, Fujian, Hunan, Guangxi, and Yunnan provinces of China [129]. *Polygala fallax Hemsl.*, a popular Chinese medicinal herb, has been used to treat various ailments, including infective inflammation and hypercholesterolemia [130].

Bai et al. demonstrated that the inhibition of NADPH oxidase-ROS-ERK1/2-NF-kB-AP-1 pathway by reinioside C reduces Ang II-induced proliferation of vascular smooth muscle cells (VSMCs) [131]. Rat aortic smooth muscle cells A10 (A10 VSMCs) were pre-treated with reinioside C (3, 10 or 30 μM), diphenyleneiodonium (DPI) a specific NADPH inhibitor (10 μM), the ERK1/2 inhibitor, PD98059 (40 μM), or the NF-κB inhibitor, pyrrolidine dithiocarbamate (PDTC) (10 μM), for 1 h before being cultured with Ang II (1 μM) for 24 h. Treatment with Ang II elevated mRNA expression of NADPH oxidase subunits (both NOX1 and NOX4) and intracellular ROS formation, and pre-treatment with reinioside C reduced significantly these effects of Ang II in a concentration-dependent manner. The phosphorylation of ERK1/2 (p-ERK1/2) elicited by Ang II was also significantly inhibited in a concentration-dependent manner after cells were pre-treated with reinioside C. Pre-treatment of the cells with DPI or the inhibitor PD98059 had the same effect. Ang II stimulation significantly increased IκBα degradation, NF-kB activity, AP-1 subunits (c-fos and c-jun), and c-myc of mRNA expression while in a concentration-dependent manner, pre-treatment of cells with reinioside C significantly reduced these effects of Ang II. Collectively, this suggest that the effect of reinioside C is by inhibition of NADPH oxidase-ROS, specifically NOX1 and NOX4 mRNA expression, and inhibition of ERK1/2-NF-κB-AP-1 pathway.

Bai et al. demonstrated oxidised low-density lipoprotein ox-(low-density lipoprotein (LDL) (100 µg/mL) for 24 h increased the adhesion of monocytes as well as elevated the expression of ICAM-1 and P-selectin in HUVECs and human monocytoid (THP-1) cells [132]. This effect by ox-LDL were attenuated by reinioside C. To determine the role of NADPH oxidase/ROS/ NF-κB pathway, NADPH oxidase subunit (NOX2 and p22phox) mRNA expression, intracellular ROS level, and NF-κB activity were measured on ox-LDL treated endothelial cells. The results revealed that reinioside C attenuated the elevated mRNA expression of NOX2 and p22phox, two NADPH oxidase subunits, reduced intracellular ROS levels and NF-kB activity caused on by ox-LDL. These findings imply that reinioside C reduces ox-LDL-induced adhesion molecule expression (P-selectin and ICAM-1) and monocyte adherence to endothelial cells via blocking the NADPH oxidase/ROS/NF-κB pathway.

### 3.5. Curcumin

Curcumin (CUR), the main ingredient of turmeric, is derived from the root of *Curcuma longa Linn* [133]. Turmeric is a spice that is made from the rhizomes of the *Curcuma longa* plant, which belongs to the ginger family (Zingiberaceae) [134]. *Curcuma longa* grows wild throughout the Indian subcontinent as well as in tropical areas, such as Indonesia and Malaysia [135]. Curcumin is still used as an alternative medicinal treatment in many parts of Southeast Asia today to cure a variety of diseases, including stomach upset [136], jaundice [137], arthritis, sprains, wounds, and skin infections [138].

Boonla et al. demonstrated CUR reduces oxidative stress and improves endothelial dysfunction and vascular remodelling in 2 kidneys 1 clip (2K-1C) hypertensive rats [139]. A model of 2K-1C renovascular hypertension in male Sprague–Dawley rats was used. The rats were treated with CUR at a dose of 50 or 100 mg/kg/day. After 6 weeks of treatment, elevated p47phox expression and higher superoxide production was observed in the vascular wall of 2K-1C rat’s aortas and carotid arteries, which was decreased with CUR treatment. Furthermore, 2K1C hypertension was associated with increased oxidative stress, which can be seen in elevated malondialdehyde (MDA) and protein carbonyl levels. CUR significantly reduced superoxide production, plasma MDA, and protein carbonyl levels in 2K-1C rats, and these negative effects were linked to a decrease in the p47phox NADPH oxidase subunit. CUR treatment also improved impaired endothelial function and this was related to increased nitrate/nitrite levels and higher eNOS expressions 2K-1C mice treated with CUR. CUR treatment prevented morphological alterations in the aorta wall found in 2K-1C hypertensive rats and significantly reduced matrix metalloproteinase-2 (MMP-2) and matrix metalloproteinase-9 (MMP-9) levels in the aortic walls generated by 2K-1C hypertension.

In the other study, CUR (30 mol/L) dramatically reduced the amount of p47phox that was overproduced by Lipopolysaccharides (LPS) in VSMCs [140]. To determine if LPS-induced cytokine upregulation in VSCMs was due to an increase in ROS, cells were pre-treated with or without DPI (20 mol/L), which inhibits NADPH oxidase before adding CUR for 1 h, and then cells were stimulated with LPS (1 g/mL) for 24 h. DPI and CUR both reduced LPS-induced Monocyte chemoattractant protein-1 (MCP-1), TNF-α, and NO upregulation. Pre-treatment of VSMCs with a combination of DPI and CUR reduced the LPS-induced rise in inflammatory cytokines synergistically. These findings imply that CUR’s anti-LPS-induced inflammatory activity is partially dependent on decreasing the formation of NADPH-mediated intracellular ROS in VSMCs.

### 3.6. Celastrol

Celastrol is one of the active compounds derived from *Tripterygium wilfordii Hook F*, a traditional Chinese medicinal plant which has been utilised for centuries [141]. Celastrol has been shown to have anti-inflammatory properties in animal models of lupus [142], Alzheimer’s disease [143], rheumatoid arthritis [144], and reduced oxidative stress injury by upregulation of Nrf2/anti-oxidant enzymes pathway [145,146].

Li M. et al. revealed that through activation of the Nrf2/ERK1/2/NOX2 signal pathway, celastrol reduces Ang II-mediated damage to HUVECs [147]. The cells were treated with arecelastrol, Ang II, Ang II + celastrol, or Ang II + celastrol+, a specific inhibitor of Nrf2 (brusatol), for 24 h. The fluorescence intensity of 2,7-dichlorodihydrofluorescein diacetate (DCFH-DA) was used to estimate intracellular reactive oxygen species generation, and NADPH oxidase activity was measured using the AmpliteTM Fluorime NADPH test Kit. At a concentration of 50 nmol/L, celastrol was able to completely inhibit the activity of NADPH oxidase and reduced the production of ROS. NADPH oxidase activity and reactive oxygen species levels in HUVECs significantly increased when co-cultured with brusatol or PD98059 (a specific Nrf2 inhibitor) compared to the celastrol. Western blotting showed celastrol significantly decreased NOX2 expression elevated by Ang II, but this effect was reversed by brusatol and PD98059. Celastrol also effectively increased anti-oxidant enzymes activities (SOD, GPx) while decreasing MDA levels in comparison to the Ang II group.

In another study, Liu et. al. demonstrated that celastrol dramatically reduced aortic valvular interstitial cell (AVIC) calcification in vitro by reducing NOX2 activity, and it greatly reduced the severity of aortic valve fibrosis, calcification, and stenosis in a rabbit model of calcific aortic valve disease (CAVD) in vivo [148]. Endogenous NOX2 knockdown reduced AVIC calcification by 39%, with an inhibitory effect similar to celastrol therapy. Celastrol therapy decreased NOX2 levels in both AVICs with osteogenic stimulation and in calcified rabbit valves, suggesting that the effect may be due to less NOX2 being induced by reduced calcification rather than by the celastrol’s direct action. Celestrol treatment also reduced NOX2 expression and ROS generation in aortic valves (AVs) of high-cholesterol (HC) plus vitamin D2-fed rabbits. Celastrol greatly decreased aortic valve ROS production, fibrosis, calcification, and severity of aortic stenosis in a rabbit CAVD model, with less left ventricular dilatation and better retained contractile function. Celastrol is useful in the treatment of CAVD, most likely through inhibiting the NOX2 in AVICs.

### 3.7. Apocynin

Apocynin (4-hydroxy-3-methoxyacetophenone) was isolated from the roots of *Apocynum cannabinum* (Canadian hemp) and was used as an approved remedy for dropsy and cardiac problems [149]. Apocynin has the ability to scavenge free radicals and act as a non-specific NOX inhibitor [150]. Apocynin has anti-inflammatory actions in atherosclerosis [151], neuroinflammatory [152], respiratory [153], and renal diseases [154] as well as cancers [155], via a ROS-dependent mechanism.

Perassa et al. demonstrated in spontaneously hypertensive rats (SHR), apocynin lowers blood pressure and restores vascular endothelial function [156]. Their results showed that in SHR, apocynin treatment significantly lowers systemic ROS. Furthermore, systolic blood pressure (SBP) and diastolic blood pressure (DBP) in apocynin-treated SHR were lower than in untreated SHR. These findings imply that reduced ROS production generated by apocynin administration may play a crucial role in the antihypertensive effect of apocynin found in several experimental hypertension models, including SHR. In SHR treated with apocynin, overexpression of NOX2 and the subunit p47phox decreased, while expression of NOX1, NOXO1, and NOX4 remains unchanged. In comparison to untreated SHR, apocynin-treated SHR reported reduced NOX2 expression. Apocynin treatment decreased the expression of p47phox in the aorta of SHR as compared to an untreated SHR. The expression of p47phox was comparable in the treated SHR and untreated Wistar rats’ aortas. The direct interaction between apocynin and p47phox prevents p47phox translocation and inhibits NOX. In SHR, apocynin improved endothelial function in the resistance and conductance arteries and increased potency to SNP in intact resistance vasculature, which may be because apocynin enhanced eNOS expression in endothelial cells. SHR aortic endothelial cells (AEC) treated with apocynin had higher [Ca^2+^] compared to untreated. Moreover, stimulation with acetylcholine doubled the amount of nitric oxide released in the AEC of treated SHR compared to untreated SHR. The ROS levels in treated SHR AEC were lower than in untreated SHR AEC.

### 3.8. Oleanolic acid

Oleanolic acid (OA) is a natural compound found in a variety of foods and medicinal plants [157]. It is a pentacyclic triterpenoid found in abundance in the *Oleaceae* family of plants, such as the olive tree [158]. Traditionally, oleanolic acid has been taken as a hepatic medication in China for over 20 years due to its hepatoprotective properties [159]. OA has been linked to antioxidant [160], antimicrobial [161], anti-inflammatory, and antidiabetic properties [162].

Jiang Q et al. reported that the anti-atherosclerosis action of oleanolic acid is facilitated by the modulation of oxidized-LDL receptor-1 (LOX1) [163]. Sixteen male quails (*Coturnix coturnix*) fed a high-fat diet and OA or simvastatin were sacrificed, and the serum was collected while the aorta was dissected out to measure NO, MDA, SOD, catalase (CAT), glutathione (GSH), NADPH, and GSH-Px levels. Their results showed that a high-fat diet significantly deteriorated the serum and aorta lipid profile as observed with increased total cholesterol, triacylglycerol, and LDL and reduced high-density lipoprotein (HDL), and treatment with OA or simvastatin reversed these lipid profile. OA and simvastatin possess an antioxidant effect, which was demonstrated with decreased MDA levels and increased GSH and NADPH levels. The activities of SOD, CAT, and GSH-px were also increased in high-fat diet quails by OA or simvastatin.

The same group also treated HUVECs with ox-LDL 200 g/mL for 24 h, with or without pre-treatment with OA (5, 10, or 20 μM) or vitamin E 20 μM for 24h and determined cell viability by MTT (3-(4,5-dimethylthiazol-2-yl)-2,5-diphenyltetrazolium bromide) tetrazolium reduction assay and ROS by DCFDA staining. Cell viability of HUVECs were decreased and production of ROS were increased after exposed by ox-LDL, which was reduced by pre-treatment with OA or vitamin E. The protein levels of gp91phox, p67phox, and p47phox (subunits of NADPH oxidase) were decreased in pre-treatment with OA after exposure to ox-LDL. Both Ho-1 and Nrf2 expression were significantly increased after 24 h of exposure to ox-LDL in HUVECs, whereas pre-treatment with vitamin E or OA further increased expression of Ho-1 and Nrf2. Additionally, LOX-1 expression was significantly increased, but pre-treatment with vitamin E or OA effectively reduced it in a concentration-dependent manner. Knockdown of LOX-1 effectively inhibited ox-LDL-induced cytotoxicity in HUVECs and also reduced ox-LDL-induced expression of Nrf2 and Ho-1 and NADPH oxidase (gp91phox, p67phox, and p47phox). These findings show NADPH oxidase may contribute to cytotoxicity, as evidenced by the fact that cytoprotection was followed by a reduction in the expression of NADPH oxidase subunits.

### 3.9. Quercetin

Quercetin (3, 5, 7, 3′, 4′-pentahydroxyflavone) is a member of the flavonoids family and one of the most prominent dietary antioxidants [164]. It can be found in various meals, including vegetables, fruits, tea, and wine, as well as a variety of healthy goods [165]. In addition, several clinical studies have shown that quercetin supplementation can help prevent and treat a variety of chronic conditions, including cardiovascular issues [166], diabetes mellitus [167], cancer [168], and obesity [169].

Galindo P et al. reported in SHRs that quercetin has cardiovascular protective effects when given orally or intraperitoneally (i.p.) [170]. SHR were randomly assigned to four experimental treatments, which are: (1) control group; (2) 10 mg kg^−1^ quercetin, single dose; (3) 10 mg kg^−1^ quercetin by oral gavage, two daily doses; and (4) 10 mg kg^−1^ quercetin i.p. injection. The results showed that when compared to normotensive Wistar–Kyoto rats, SHR aortas showed significantly reduced endothelium-dependent relaxation (EDR) and nitric oxide (NO)-dependent vasodilator responses to ACh in arteries stimulated by phenylephrine, which was reversed with chronic oral quercetin administration. The aortic superoxide formation was also decreased with chronic oral quercetin treatment. protein expression of the NADPH subunits p47phox, NOX1, and NOX4 were reduced by quercetin treatment in the aorta via oral, but via i.p. administration. Intraperitoneal quercetin treatment failed to ameliorate endothelial dysfunction and reduced both NADPH oxidase activity and protein expression of any NADPH oxidase subunits (p47phox, NOX1, and NOX4), suggesting that oral quercetin was superior to intraperitoneal administration for the protection from cardiovascular complications in SHR.

Wan et al. explored the effects of quercetin on NOX2, eNOS, and inducible NOS (iNOS) after myocardial ischemia-reperfusion injury (MIRI) in rabbits [171]. NOX2 mRNA expression was higher in I/R hearts after 30 min of coronary ligation followed by 12 h of reperfusion, as determined by real-time PCR, compared to control hearts. Western blotting tests, which provide a real assessment of protein expression, were also done to explore the relative expression of NOX2 in both strains, and NOX2 protein expression was found to be significantly increased in I/R hearts. NOX2 mRNA and protein expression in I/R hearts following quercetin administration were lower in I/R + quercetin rabbits than in I/R rabbits. Similarly, NOX2 mRNA and protein levels in quercetin treated animals were significantly lower than in control rabbits. Quercetin suppressed not only NOX2 but also iNOS mRNA, eNOS mRNA, and protein expression induced by MIRI [171]. The current study provides the first evidence that quercetin, a flavonoid, inhibits cardiac ischemia-reperfusion-induced NOX2, iNOS, eNOS mRNA, and protein expression. As a result, quercetin could be a potential antioxidant for cardioprotection.

### 3.10. Delphinidin-3-Glucoside

Delphinidin, a prominent anthocyanins found in pigmented fruits and vegetables such as pomegranates, berries, dark grapes, eggplant, tomatoes, and carrots, is one of the most common anthocyanins [172]. Delphinidin is a potent radical scavenger capacity for superoxide [173]. Delphinidin has been shown to have anti-inflammatory [174], antioxidant [175], anticancer [176], cardiovascular protection [177], and neuroprotection [178] properties.

Xie et al. demonstrated delphinidin-3-glucoside effects on ox-LDL-induced apoptosis and oxidative stress in cultured porcine aortic endothelial cells (PAEC) [179]. PAEC were treated with ox-LDL, delphinidin alone, and a co-treatment of delphinidin and ox-LDL. Their findings revealed the amount of intracellular superoxide in PAEC rose more than two-fold after a 2 h treatment with 100 μg/mL ox-LDL as measured using 2,7-dichlorodihydrofluorescein diacetate (H2DCF-DA) and enhanced lucigenin assay, respectively. Compared to the control, delphinidin alone at 100μM decreased intracellular superoxide by more than 50%. Compared to ox-LDL alone, co-treatment with delphinidin and ox-LDL reduced the amount of superoxide elevated from ox-LDL in endothelial cells. Moreover, increased levels of NOX2, NOX4, p22phox, and caspase 3 produced by ox-LDL in PAEC were decreased by co-treatment with delphinidin. The data suggest that ox-LDL caused oxidative stress and apoptosis in endothelial cells, which was related with NOX activation, mitochondrial respiration chain enzyme impairment, and the disruption of critical apoptosis regulators.

Chen et al. evaluated the effect of delphinidin in a model of cardiac hypertrophy using the transverse aortic constriction (TAC)-induced pressure overload method and primary culture of neonatal rat cardiomyocytes. The result showed that delphinidin reduces pathological cardiac hypertrophy by suppressing the NOX/MAPK signalling pathway to counteract oxidative stress [180]. In comparison to the sham control group, there was also an increase in ROS levels and NOX activity. However, delphinidin at the high dosage (15 mg/kg/day) prevented these changes. In Ang II-induced hypertrophy of neonatal rat cardiomyocytes (NRCMs), the NOX subunit proteins p22phox, p47phox, p40phox, p67phox, and gp91phox were all considerably upregulated by Ang II stimulation, while delphinidin coadministration inhibited the upregulation of p47phox. Compound C, an AMPK inhibitor, prevented the delphinidin-mediated upregulation of Rac1 and lowered expression levels of p47phox. The expression of pathogenic genes, the formation of ROS, and NOX activity were all raised by compound C, which also removed the delphinidin-mediated reduction of the Ang II-induced hypertrophy of neonatal rat cardiomyocytes (NRCMs). Therefore, these findings revealed that delphinidin activated AMPK, which inhibited ROS production, particularly p47phox, and pathological hypertrophy by downregulating NOX.

**Table 2:** List of natural products that target the NADPH oxidase pathway in CVD. The arrow 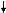 indicate down-regulation and 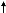 indicate up-regulation of the markers.

Natural Product	Plant	Effective Dosage	Induction of Cardiovascular Condition	Model	Result	NADPH Oxidase Subunit Involved	Reference
Berberine 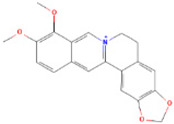	*Rhizoma Coptidis*	10 μM	Endothelial microparticles (EMPs)-induced endothelial dysfunction	Human study In vitro using HUVECs	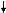 ROS, NOX4, NO, MDA, CD31+/CD42 microparticles	NOX4	[105]
		5.0 μmol/L	Palmitate-induced endothelial dysfunction	In vitro using HUVECs	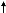 eNOS, AMPK, NOX4	NOX4	[106]
Paeonol 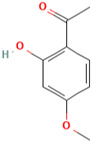	*Paeonia suffruticosa*	20 mg/kg/day	Tunicamycin-induced endothelial dysfunction	In vivo	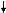 ROS, GRP78, ATF6 and p-eIF2α, NOX2 eNOS, NO	NOX2	[115]
		100 mg/kg/day	Methotrexate (MTX)-induced	In vivo	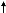 eNOS, NO, GSH, SOD 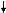 NOX2, MDA, TLR4	NOX2	[116]
Thymoquinone 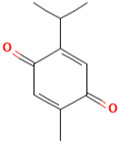	*Nigella sativa*	50 mg/kg/day 20 µmol/L	Angiotensin II-induced cardiac damage	In vivo In vitro (H9c2 cells)	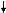 IL-1, IL-6, TNF, collagen I, collagen III NOX4 and p53	NOX4	[125]
		100 mg/kg/day	Sepsis-induced cardiac damage	In vivo	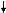 IL-6, TNF, Bax, NOX4, p-PI3K, and p-AKT 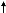 Bcl-2, Ho-1, and Nrh2 expression	NOX4	[126]
		50 mg/kg/day 5 µM	Transverse aortic constriction	In vivo In vitro (neonatal rat cardiomyocytes)	mRNA levels of NOX4, SOD1, SOD2 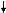	NOX4	[127]
Reinioside C 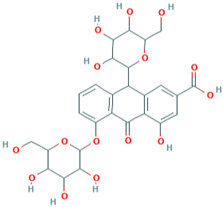	*Polygala fallax Hemsl.*	30 µM	Arteriosclerosis	In vitro (VSMCs)	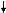 ROS, NOX1, NOX4, IB degradation, NF-kB activity, and p-ERK1/2	NOX1 NOX4	[131]
		10 µM	Atherosclerosis	In vitro (HUVECs, THP-1)	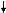 NOX2 and p22phox, ICAM-1, P-selectin, NF-κB	NOX2 and p22phox	[132]
Curcumin 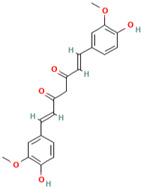	*Curcuma longa Linn*	100 mg/kg/day	Renovascular hypertension	In vivo (2K-1C model) In vitro (VSMCs)	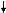 MMP-2 and MMP-9, ROS, MDA and p47phox 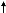 EDR, eNOS	p47phox	[139]
		30 μmol/L	Atherosclerosis	In vitro (VSMCs)	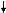 /> p47phox, MCP-1, TNF	p47phox	[140]
Celastrol 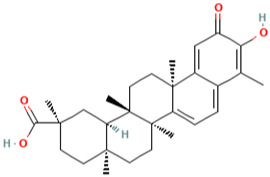	*Tripterygium wilfordii Hook F*	50 nmol/L	Hypertension	In vitro (HUVECs)	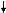 MDA, NOX2 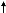 SOD and GPx-SH	NOX2	[147]
		1 mg/kg/day 10 nmol/L	Calcific aortic valve disease	In vivo (rabbit) In vitro (AVICs)	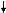 NOX2	NOX2	[148]
Apocynin 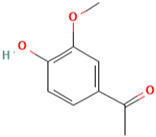	*Apocynum cannabinum*	(30 mg/kg)	Hypertension	In vivo (SHR rats) In vitro (endothelial cells)	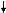 ROS, MAP, HR, SBP, DBP, NOX2 and p47phox 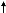 eNOS, cytosolic calcium, and NO	NOX2 and p47phox	[156]
Oleanolic acid 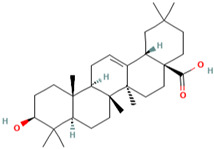	*Oleaceae*	100 mg/kg/day 20 µM	Atherosclerosis	In vivo (high-fat diet-induced atherosclerosis in quails) In vitro (HUVECs)	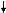 MDA, GSH, gp91phox, p67phox and p47phox, LDL 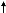 HDL, SOD, CAT GPx-SH, LOX-1, Nrf2 and ho-1	gp91phox, p67phox and p47phox	[163]
Quercetin 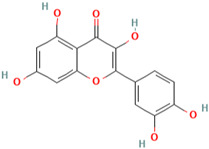	Berries, onions, and red wine	10 mg/kg/day	Hypertension	In vivo (SHR)	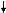 SBP, HR, NOX1, NOX4, p47phox EDR	NOX1, NOX4, p47phox and p22phox	[170]
		1 mg/kg	Myocardial ischemia-reperfusion injury	In vivo (rabbit)	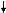 NOX2, eNOS, iNOS	NOX2	[171]
Delphinidin 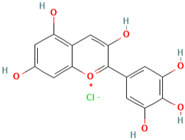	Pigmented fruits and vegetables	100 μM	Hypercholesterolemia	In vitro (ox-LDL-induced ROS in PAEC)	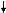 NOX2, NOX4, and p22phox, caspase 3	NOX2, NOX4, and p22phox	[179]
		15 mg/kg/day 50 μM	Cardiac hypertrophy	In vivo (TAC-induced pressure overload C57BL/6 mice) In vitro (neonatal rat cardiomyocytes)	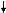 p47phox	p47phox	[180]

## 4. Conclusions

A variety of natural bioactive compounds have been shown to possess potent medicinal properties in treating and preventing cardiovascular diseases. It is demonstrated that natural bioactive compounds provide an advantage as a protective agent or supplementary or combination therapy against cardiovascular diseases. Our review concludes that various natural bioactive compounds provide protection against cardiovascular diseases by targeting the NADPH oxidase pathway. These include: berberine targeting NOX4; paeonol targeting NOX2; thymoquinone targeting NOX4; reinioside C targeting NOX1,2,4, and p22phox; curcumin targeting p47phox; celastrol targeting NOX2; apocynin targeting NOX2 and p47phox; oleanolic acid targeting gp91phox, p67phox, and p47phox; quercetin targeting NOX1,2,4, p47phox, and p22phox; and delphinidin targeting NOX2, NOX4, p22phox and p47phox. However, there are limited clinical, pharmacokinetic, and pharmacodynamic studies of these natural bioactive compounds; thus, restricting the potential use of the natural bioactive compounds for supplementary treatment and management plans for patients with cardiovascular diseases. Future research in both fundamental, pharmacokinetic, or pharmacodynamics profile and clinical trials are warranted to explore the underlying mechanism and the effectiveness of those listed natural bioactive compounds to prevent or reduce the occurrence of cardiovascular diseases, specifically coronary artery disease.
